# Application of New Al-Si Welding Filler with High Concentration of Copper and Magnesium: High-Temperature Strength and Anti-Corrosion Mechanism

**DOI:** 10.3390/ma17010126

**Published:** 2023-12-26

**Authors:** Jun-Ren Zhao, Fei-Yi Hung, Chien-Yu Pan

**Affiliations:** Department of Materials Science and Engineering, National Cheng Kung University, Tainan 701, Taiwan; a2x346yz03@gmail.com (J.-R.Z.);

**Keywords:** Al-Si-Cu-Mg alloy (SCM), welding, microstructure, mechanical properties

## Abstract

Currently, the primary commercial aluminum alloy fillers used are 4043 and 5356. However, when welded with high-strength work pieces like 6061 and 7075 aluminum alloys, the strength of weld beads significantly lags behind that of the original welded material. Both 4043 and 5356 aluminum alloys cannot be strengthened through heat treatment. The strength difference between the weld bead and base material doubles after heat treatment. In this study, an Al-Si-Cu-Mg alloy (SCM) filler modified using a heat-treatable A319 aluminum alloy was employed to investigate the post-welding microstructural and mechanical properties of specimens under room- and high-temperature conditions and after prolonged exposure in a saltwater environment (3.5 wt.% NaCl). The aim was to demonstrate that commercial aluminum alloy fillers could be substituted with a high-silicon aluminum alloy boasting excellent solidification and mechanical properties. The results revealed that, after heat treatment of the weld bead, dendrites were not eliminated, but the tensile strength increased to 310 MPa, closely matching that of commercial 6061 aluminum alloy. The strength of the weld bead remained higher than 250 MPa in high-temperature (240 °C) and saltwater environments. These findings underscore the potential application of this material.

## 1. Introduction

Tungsten inert gas (TIG) welding is an arc welding process where the metal is heated by the arc between the electrode and the welded metal, resulting in metal agglomeration. This process maintains a stable arc and ensures adequate welding quality [1,2,3]. TIG welding is commonly used for various materials, including stainless steel [4,5], magnesium alloys [6,7], and aluminum alloys. Among aluminum alloys, TIG welding finds widespread application not only in the 2XXX series [8] and 7XXX series [9] but is most commonly employed in the 6XXX series [10,11,12]. With significant advancements in the transportation industry, particularly in aviation, fillers such as 4043 and 5356 aluminum alloys are extensively used for welding to higher-strength alloys like 6061 or 7075 [13,14]. However, the use of 4043 or 5356 alloys poses challenges, including reduced strength, corrosion resistance, and instability at high temperatures [3]. This has led to a growing demand for the development of fillers with enhanced strength, corrosion resistance, and stability at high temperatures.

The 6061 Al alloy plate used in this study is a heat-treatable aluminum alloy that can be strengthened by the precipitation of Mg_2_Si through a solution treatment and aging heat treatment [15,16,17,18,19,20]. Ding et al. [16] demonstrated that the addition of Zn could increase the precipitation behavior. Zheng et al. [20] studied the influence of different Si content and confirmed that an increase in Si content could improve the age-hardening response. Chakrabarti and Laughlin [18] revealed that the Al–Mg–Si–Cu alloy contains the common quaternary phase Q. Ozturk et al. [19] studied the different aging conditions of 6XXX aluminum alloy, and the results showed that different Mg/Si atomic ratios would lead to different times to peak age. The filler for the 6061 aluminum alloy plate is a Si-Cu-Mg (termed SCM) aluminum alloy modified from A319. To improve the fluidity of the welding rod after melting and reduce welding cracks, the concentration of Si was increased from 5.5–6.5 wt.% in A319 to 8.8 wt.% in the SCM aluminum alloy, thereby reducing the solidification shrinkage rate [21].

To date, almost all studies on the use of 4356 and 5356 aluminum alloys as fillers for welding to 6061 aluminum alloy have focused on the mechanical properties at room temperature, whether using TIG welding [22,23,24] or laser beam welding [25,26]. Due to the strength gap between the weld bead and the substrate plate at room temperature, there have been few studies further investigating the high-temperature mechanical properties of weld beads. In corrosion resistance research, Fahimpour et al. [27] and Öteyaka et al. [28] have investigated and compared the polarization curves for FSW and TIG welding of 6061 aluminum alloy immersed in a 3.5 wt.% NaCl solution. Mutombo et al. [29] investigated the difference in fatigue properties between 6061 aluminum alloy samples exposed to an air atmosphere or immersed in a 3.5 wt.% NaCl solution. All the mentioned studies have shown that after immersion in a 3.5 wt.% NaCl solution, 6061 aluminum alloy has a lower corrosion potential and poorer corrosion resistance than before immersion. However, studies investigating changes in the alloy’s mechanical properties after immersion in a 3.5 wt.% NaCl solution are lacking.

In this study, a SCM aluminum alloy filler was welded to a 6061 aluminum alloy substrate plate through TIG welding to investigate the microstructural and mechanical properties of the original filler. Additionally, the study examined the element distribution, microhardness, and microstructural mechanical properties of the weld bead after welding. The influence of heat treatment on the microstructural and mechanical properties of the weld bead was discussed. Finally, the research delved into the mechanical changes in the heat-treated weld bead under high-temperature and saltwater environments, providing valuable application data for this SCM aluminum alloy filler. This data can facilitate the filler’s application in structures such as ships and boilers that require bilateral welding and high-temperature operation.

## 2. Experimental Procedure

**Figure 1:**
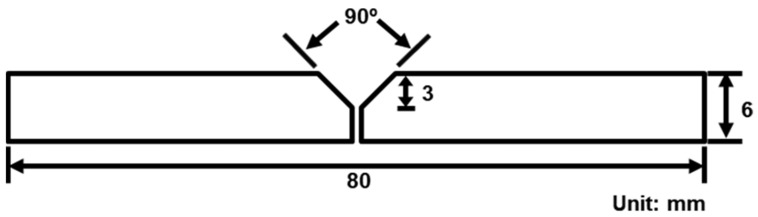
Schematic of the welded substrate plate chamfer.

**Figure 2:**
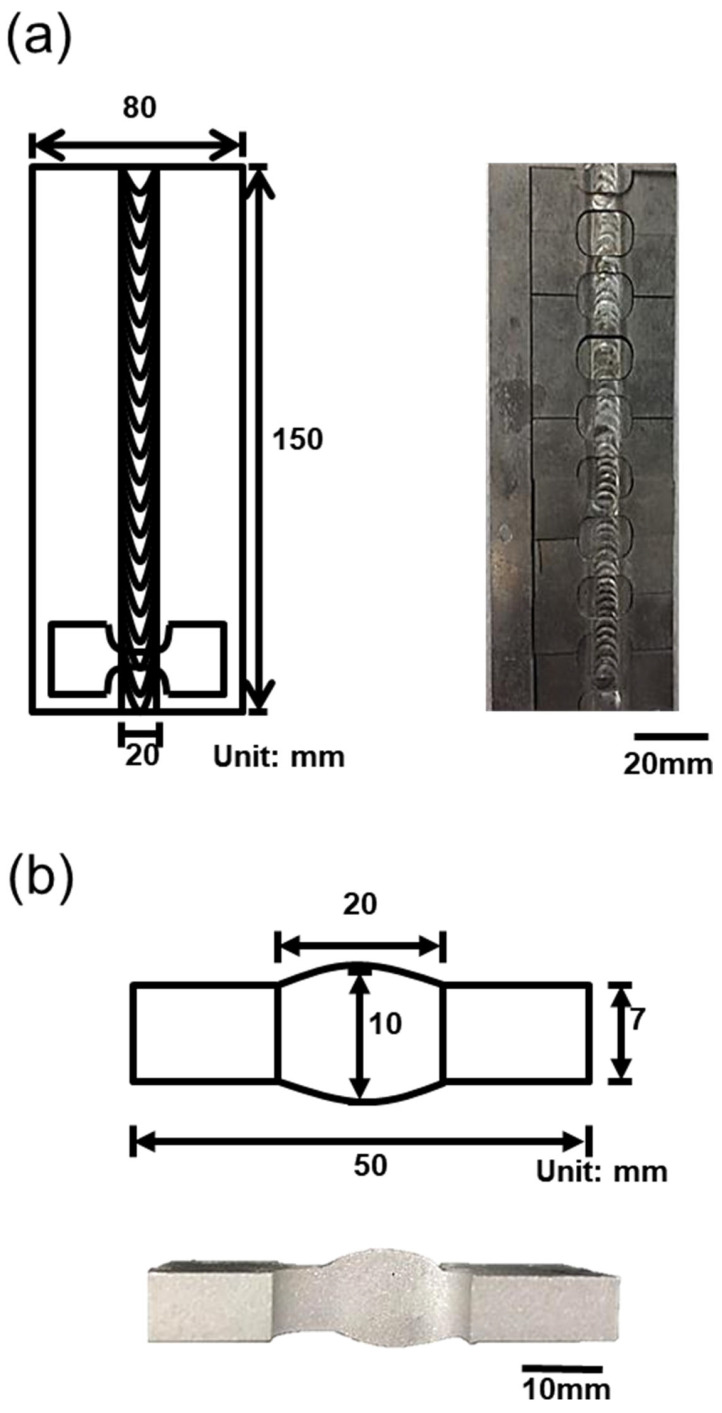
Welded specimen: (**a**) top view, (**b**) side view.

**Figure 3:**
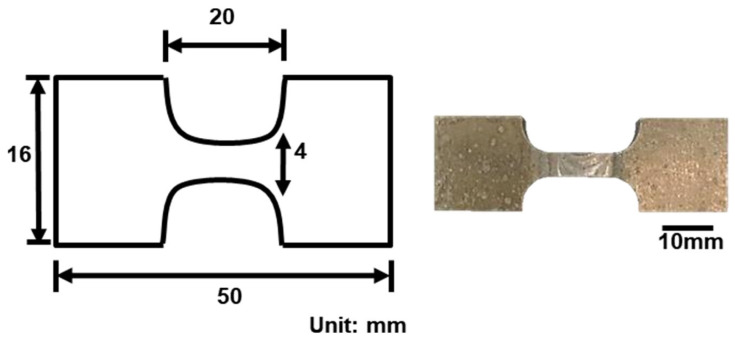
Schematic of the welded tensile specimen.

A Si-Cu-Mg aluminum alloy filler modified from A319 was used as the experimental material. The comparison of the chemical composition of the SCM filler, A319, and 6061 plate are listed in Table 1. The chemical composition includes Si (8.8 wt.%), Cu (3.9 wt.%), Mg (0.35 wt.%), Mn (0.26 wt.%), and trace amounts of Fe, Cr, and Zr (approximately 0.1 wt.%), classifying it as an aluminum alloy with high concentrations of Si and Cu elements. Subsequently, the welding rod, drawn to a diameter of 3.2 mm, was used for the bilateral TIG welding of a 6061 aluminum alloy plate (thickness: 6 mm) with a 45° chamfer. The TIG welding process parameters used in this study included a 3 mm electrode-to-workpiece distance, a 10 L/min argon gas flow rate, 15 V voltage, 5 mm/s welding speed, and 30 A AC. The chamfering diagram is presented in Figure 1. Figure 2 depicts a schematic of the specimen after welding, which was subsequently cut into a weld bead tensile specimen through wire cutting (as illustrated in Figure 3). According to a previous study [30], aluminum alloys with high Si and Cu concentrations should undergo solution heat treatment at a lower temperature. For this study, the chosen conditions were 470 °C for 6 h with water cooling for the solution treatment and 170 °C for 6 h with water cooling for the artificial aging. The weld bead, after undergoing heat treatment, is denoted by the code SCM-T4/T6.

First, #100 to #4000 SiC sandpaper was used to grind the specimens. Subsequently, a polishing solution containing 1 μm, 0.3 μm of Al_2_O_3_, and 0.04 μm of SiO_2_ was applied for polishing. Next, Keller’s reagent etching solution (20 mL of HNO_3_, 15 mL of HCl, 10 mL of HF, and 60 mL of H_2_O) was used to etch the welding rod and weld bead. An optical microscope (OM, OLYMPUS BX41M-LED, Tokyo, Japan) was utilized to observe the specimen’s microstructure and explore the differences in the welding zone (WZ), partial melting zone (PMZ), and heat-affected zone (HAZ) of the specimen. X-ray diffraction (XRD) spectroscopy (Bruker AXS GmbH, Karlsruhe, Germany), scanning electron microscopy (SEM, HITACHI SU-5000, HITACHI, Tokyo, Japan), and energy dispersive spectroscopy (EDS) were employed to analyze the specimen’s second-phase morphology and distribution. Electron probe microanalysis (EPMA, JEOL JXA-8900R, Taipei, Taiwan) was employed to investigate the distribution of alloy elements.

**Figure 4:**
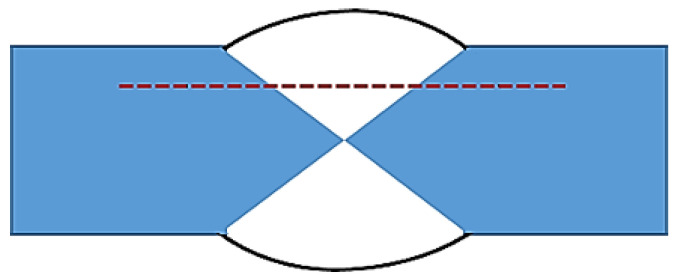
Schematic of the microhardness test path (dotted line).

A micro-hardness machine (Shimadzu HMV-2000L, Shimadzu, Kaohsiung, Taiwan) was used to investigate the hardness of the weld bead; the test method is shown in Figure 4. The welding plate was tested every 0.5 mm along a straight line. The center of the straight line was the center of the weld, and the total test length was 10 mm. A universal testing machine (HT-8336, Hung Ta, Taichung, Taiwan) was used for the tensile test. The initial tensile strain rate was 1.67 × 10^−3^ s^−1^, and the result was the average of three test results. A scanning electron microscope and optical microscope (OM) were used to examine specimen tensile fracture characteristics under each experimental condition.

To investigate the high-temperature mechanical properties of the weld bead after heat treatment, a universal testing machine was used to conduct a tensile test at 120 °C, 180 °C, and 240 °C, and the initial tensile strain rate was 1.67 × 10^−3^ s^−1^ (the tensile test result was the average of three tests results). Additionally, the specimen was immersed in a 3.5 wt.% NaCl solution maintained at 36 °C for 1 day, 3 days, and 7 days to clarify the degradation rate of the mechanical properties of the heat-treated weld bead after chlorination. Finally, after the specimen was immersed in a 3.5 wt.% NaCl solution at 60 °C for 2 days, the cross-section of the corroded specimen was observed with an OM to clarify the corrosion mechanism.

## 3. Results and Discussion

### 3.1. Microstructural and Element Distribution of SCM Weld Zone

**Figure 5:**
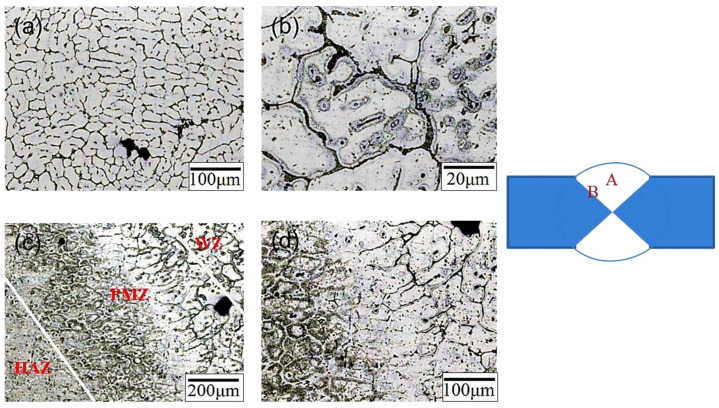
Microstructure of the SCM-AW weld zone: (**a**) low- magnification image of position A, (**b**) high-magnification image of position A, (**c**) low-magnification image of position B, and (**d**) high-magnification image of position B.

Figure 5 shows the microstructure of the original SCM weld bead (code SCM-AW). The microstructure of the center of the weld bead (position A) is shown in Figure 5a,b; the indicated structures were α-Al dendrites with microstructural similarity to that of traditional cast A319 [31,32]. High-magnification [Figure 5b] observation revealed the secondary phase precipitates in the interdendritic areas. Figure 5c,d show the microstructure (position B) at the junction of the weld bead and the substrate, which can be divided into three areas, namely the welding zone (WZ), partial melting zone (PMZ), and heat-affected zone (HAZ). During welding, the molten SCM aluminum alloy was in contact with the 6061 substrate plates, and the solidification rate in the WZ was extremely fast (10^−3^ s^−1^*)*, producing columnar dendrites parallel to the direction of heat flow. Along the direction of the substrate plate is the PMZ, where the columnar dendrites in the direction of heat flow are relatively irregular. The outermost region is the HAZ, where no columnar dendrites are arranged along the direction of heat flow. The HAZ underwent a similar process to heat treatment; it was affected by high temperatures during welding.

**Figure 6:**
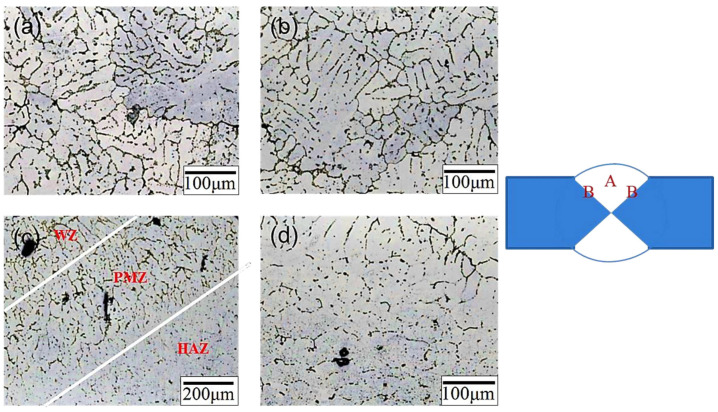
Microstructure of the SCM-T4/T6 weld zone: (**a**) low-magnification image of position A, (**b**) low-magnification image 2 of position A, (**c**) low-magnification image of position B, and (**d**) high-magnification image of position B.

**Figure 7:**
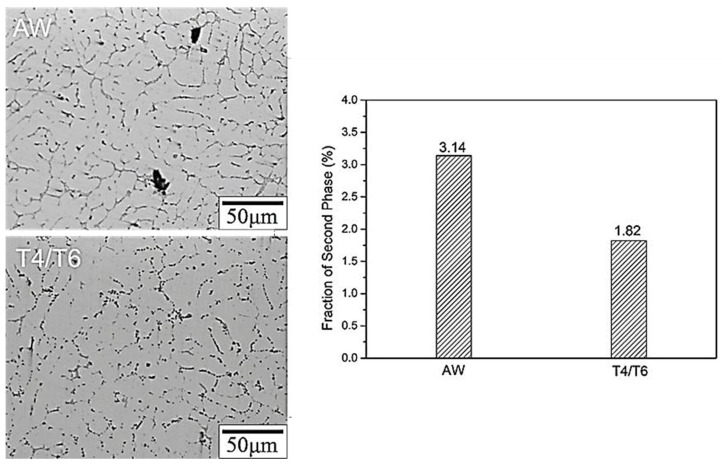
Percentage of secondary phases particles before and after heat treatment of the SCM weld zone.

Figure 6 illustrates the microstructure of the weld bead after T4/T6 heat treatment. The microstructure was approximately the same as it was before heat treatment, but the dendrites in the center of the weld bead (position A, Figure 6a,b) became larger. The microstructure at the junction of the weld bead and the substrate plate (position B, Figure 6c,d) also exhibited a more uniform arrangement than before heat treatment. This microstructure was similar to that of 4043 used as a filler for welding to 6061 aluminum alloy [24,33]. A comparison of the percentage of secondary phase particles before and after heat treatment of the SCM weld bead is presented in Figure 7. The percentage was 3.14 % before heat treatment and 1.82 % after T4/T6 heat treatment, confirming the effectiveness of the solid-solution treatment.

**Figure 8:**
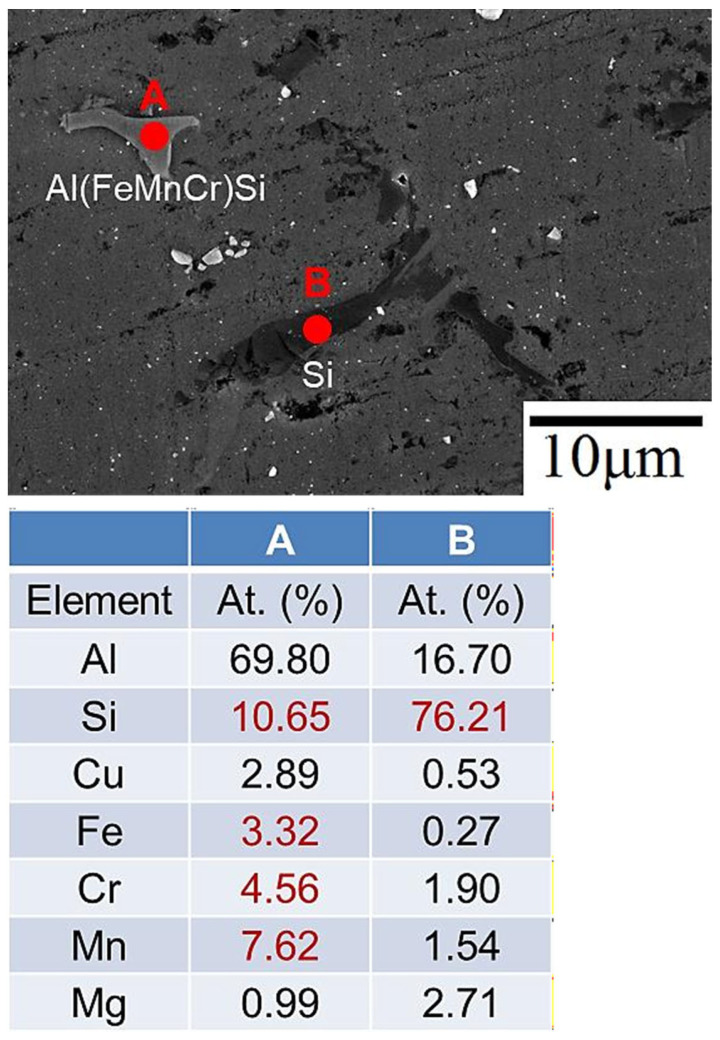
EDS analysis results for the SCM-AW weld zone.

**Figure 9:**
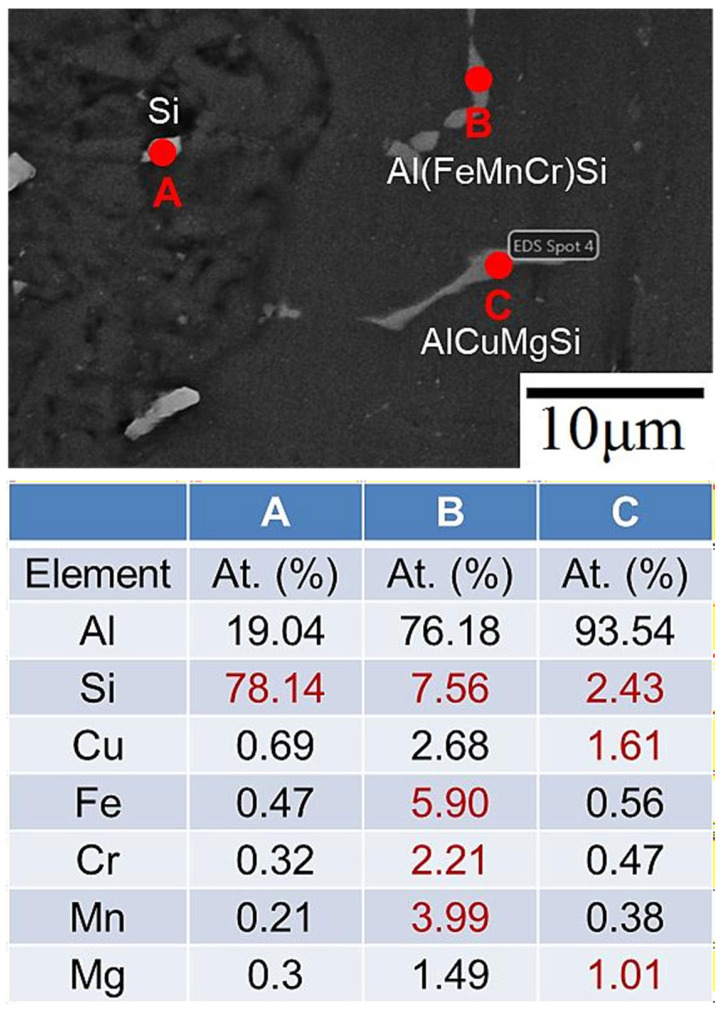
EDS analysis results for the SCM-T4/T6 weld zone.

**Figure 10:**
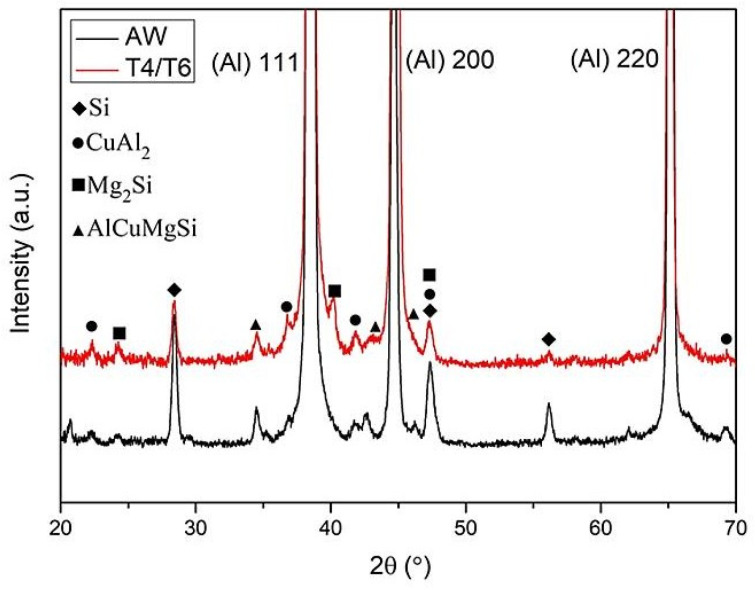
XRD analysis results for the SCM weld zone before and after heat treatment.

XRD, SEM-EDS, and EPMA were used to determine the type and morphology of the crystalline phase before and after heat treatment. According to one study [34], for an aluminum alloy whose chemical composition is similar to that of an SCM filler, the secondary phases mainly include Si particles, Al_2_Cu, Mg_2_Si, Al_5_Mg_8_Si_6_Cu_2_, and Al_5_FeSi phases. The EDS analysis results for the weld bead before and after heat treatment are shown in Figure 8 and Figure 9, respectively. Figure 8 displays the weld bead before heat treatment. At point A, there is a higher atomic percentage of Si, Fe, Cr, and Mn elements, speculated to be the Al(FeMnCr)Si phase. At point B, a prominent Si element atomic percentage is observed, presumed to be Si particles. Figure 9 illustrates the weld bead after heat treatment. At point A, there are also more Si elements, representing Si particles, while at point B, there are higher atomic percentages of Si, Fe, Cr, and Mn elements, indicating the Al(FeMnCr)Si phase. Additionally, point C exhibits higher atomic percentages of Si, Cu, and Mg elements, speculated to be the Al-Cu-Mg-Si phase. Al-Fe-Mg-Si is a high-temperature phase, which means that it cannot be dissolved in the matrix after heat treatment. Figure 10 shows the XRD analysis results for the weld bead before and after heat treatment. In addition to the aforementioned phases, the Al_2_Cu phase was detected in the XRD analysis. According to a related study [17], because of the small size of the A_l2_Cu phase, it is difficult to verify its presence using SEM-EDS. Furthermore, the peak value of Si drops considerably after heat treatment.

**Figure 11:**
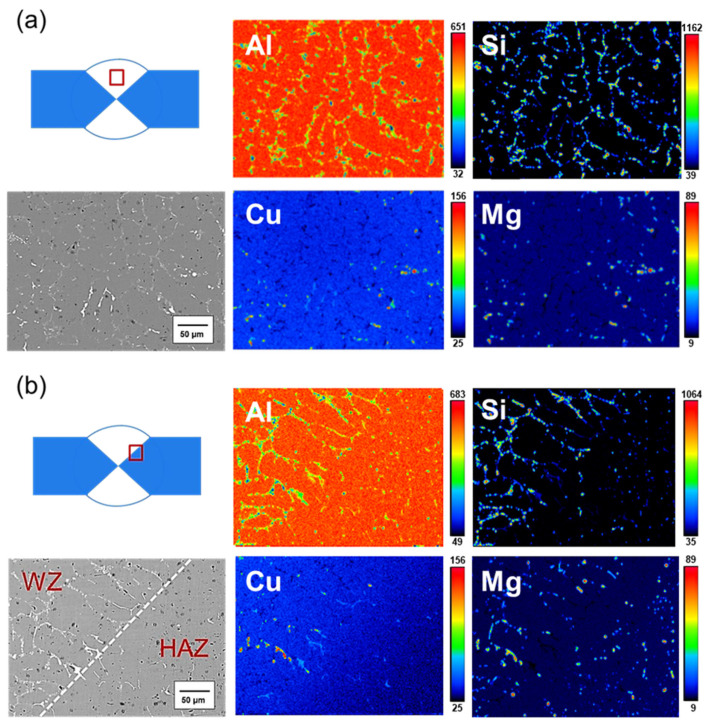
EPMA results for the SCM-AW specimen: (**a**) center of the weld bead, and (**b**) junction of the weld zone.

**Figure 12:**
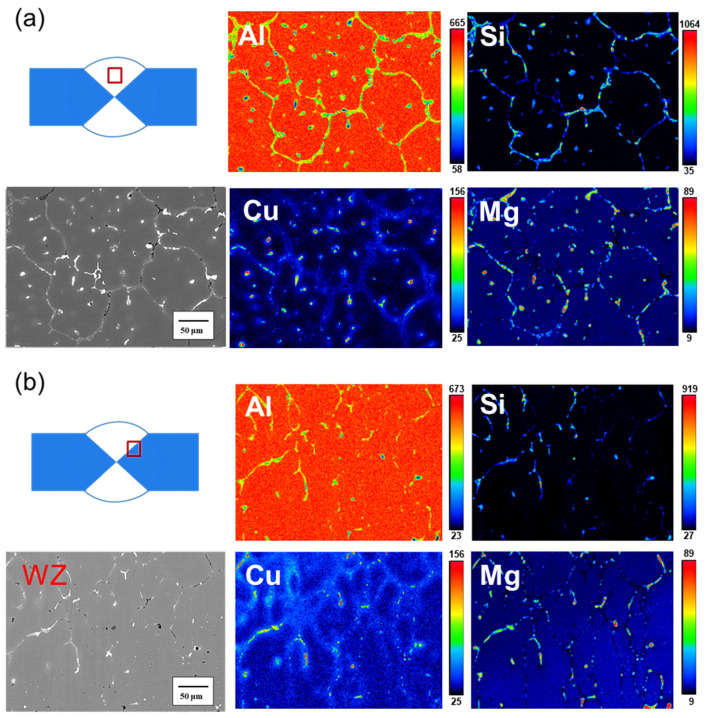
EPMA results for the SCM-T4/T6 specimen: (**a**) center of the weld bead, and (**b**) junction of the weld zone.

Figure 11a displays the EPMA results for the center of the weld bead before heat treatment. The figure illustrates Si distribution at the grain boundary, with a weak Si signal in the white particles at the grain boundary in the SEM image. Based on the earlier EDS and XRD phase analysis results, this white particle phase could be Al-Fe-Mn-Si or Al-Cu-Mg-Si. The phase where Cu and Mg signals overlap is identified as Al-Cu-Mg-Si. In Figure 11b, the EPMA results for the junction of the weld bead and the Heat-Affected Zone (HAZ) before heat treatment are presented. The Si signal is predominantly distributed at the grain boundary. Compared to the weld bead, the signal overlap between Mg and Si is lower at this junction, indicating that most of the Si segregated in the grain boundary is in the Si particle phase, and some Si particles are in the Mg_2_Si phase. The Cu signal mainly appears in the grain boundary of the weld bead area, with almost no Cu signal in the HAZ, owing to the difference in chemical composition between the 6061 substrate plate and SCM filler. Figure 12a showcases the EPMA results for the center of the weld bead after heat treatment. The gray phase in the grain boundary is primarily where the Si signal alone is strong, and the white phase in the grain boundary is the Al-Fe-Mg-Si or Al-Cu-Mg-Si phase formed by Si and other elements. The overlap of Mg and Cu signals is likely the Al-Cu-Mg-Si phase, consistent with the earlier EDS analysis results (Figure 9). In Figure 12b, the EPMA results for the junction of the weld bead and the HAZ after heat treatment are presented. The Si signal is still concentrated at the grain boundary, but Si now overlaps with Mg and Cu at the grain boundary. While Si was a separate particle phase before heat treatment, it combined with other elements to form a phase after heat treatment.

### 3.2. Mechanical Properties of SCM Weld Zone in Different Environments

**Figure 13:**
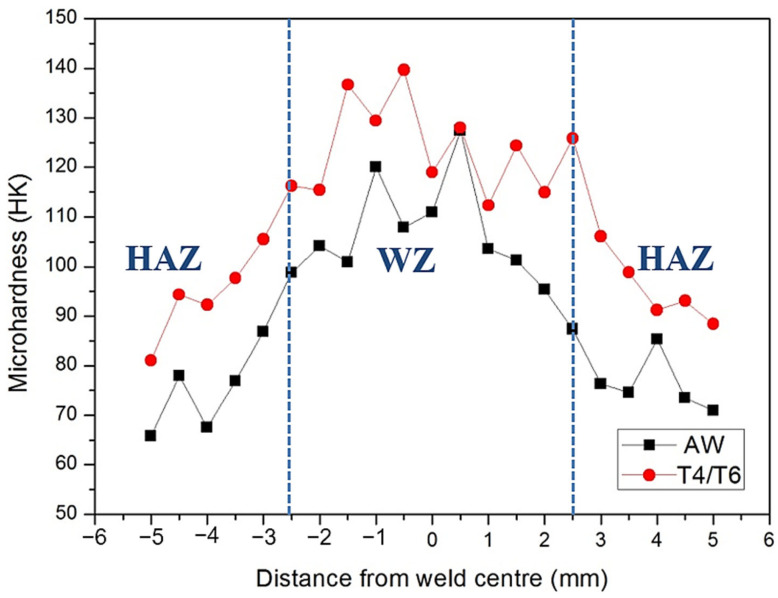
Microhardness distribution of the SCM weld zone before and after heat treatment.

The microhardness distribution of the SCM weld bead is depicted in Figure 13. Before heat treatment, the center of the weld bead exhibited the highest hardness, with lower values at the sides, and the Heat-Affected Zone (HAZ) showed the lowest hardness. The presumed location of the tensile fracture was in the HAZ. Following heat treatment, there was an overall increase in hardness, and the microhardness distribution trend remained unchanged. This observation contrasts with the findings of Fauzi et al. [10], who utilized 4043 as a filler, and Abbass et al. [35], who employed 5356 as a filler. In those studies, the microhardness values of the weld beads were lower than those of the substrate plate. In contrast, the SCM weld bead in this study exhibited higher microhardness than the substrate plate.

**Figure 14:**
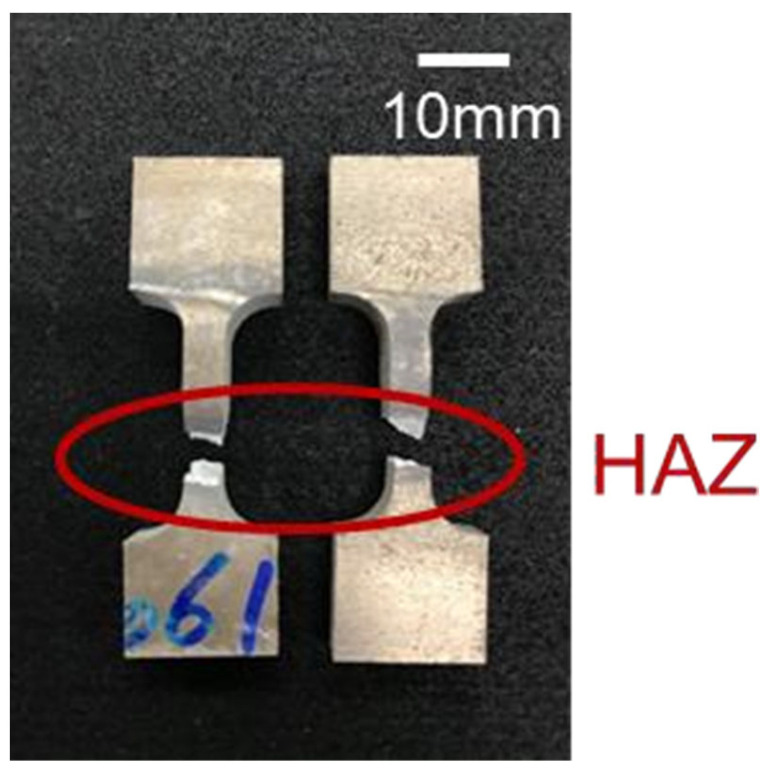
Macroscopic photograph showing the morphology of fractured SCM-T4/T6 tensile fracture specimens.

Table 2 provides a comparison of the mechanical properties of the original SCM filler and the welding specimen before and after heat treatment. The strength and ductility of the SCM filler showed a significant increase after heat treatment. When compared to the commercial 6061 alloy after T6 treatment [36], this specimen exhibited markedly superior yield strength (YS) and ultimate tensile strength (UTS), while maintaining ductility above a certain threshold [19,37,38]. This indicates that the heat treatment effectively enhanced the mechanical properties of the specimen. Although the strength of the specimen after welding was initially lower than that of the original filler, it increased to over 300 MPa after heat treatment, still surpassing that of commercial 6061 plates subjected to T4/T6 heat treatment. Notably, irrespective of heat treatment, the strength of this SCM filler after welding exceeded the maximum tensile strength (140 MPa) of the 5356 aluminum alloy filler [39]. Figure 14 illustrates the macro-morphology of the fractured SCM-T4/T6 tensile specimen. As depicted, all fractures were located in the Heat-Affected Zones (HAZs), aligning with the microhardness distribution presented in Figure 13.

**Figure 15:**
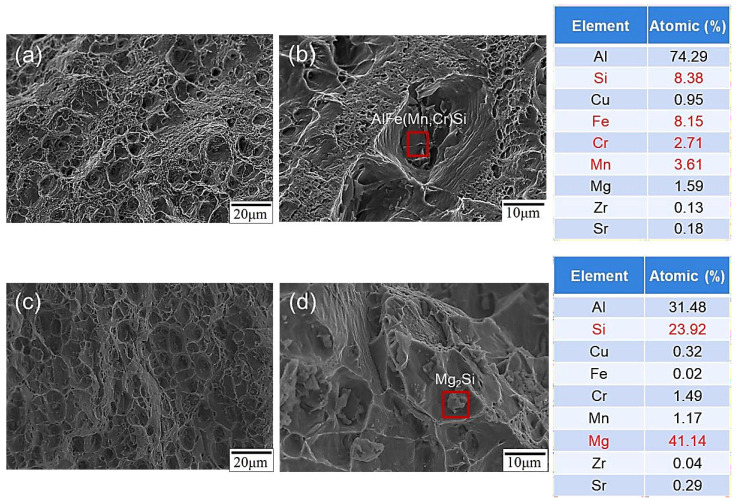
Morphologies of the SCM weld zone before and after heat treatment of tensile fracture surfaces: (**a**) low-magnification image of SCM-AW, (**b**) high-magnification image of SCM-AW, (**c**) low-magnification image of SCM-T4/T6, and (**d**) high-magnification image of SCM-T4/T6.

When subjected to tensile, the specimen fractured in the region characterized by lower hardness. Figure 15 displays the microstructure of the fractured section of the SCM weld bead both before and after heat treatment. Following heat treatment, the dimple-like morphology of the specimen was less pronounced, suggesting reduced ductility; this inference was supported by corresponding tensile data (see Figure 15c). According to the EDS analysis results of the second phase particles with dimple-like morphology before and after heat treatment, as shown in Figure 15b,d, the fractured particles before heat treatment exhibit higher contents of Fe, Mn, Cr, and Si elements. It is inferred that these particles are hard and brittle AlFe (Mn, Cr) Si phase particles. On the other hand, the fractured particles after heat treatment contain higher contents of Mg and Si elements, indicating that they are Mg_2_Si phase particles. As mentioned in a previous section, the fracture locations were all in the Heat-Affected Zones (HAZs) within the dominant region of the 6061 aluminum alloy substrate. Consequently, the majority of the fractured particles were identified as Mg_2_Si phase ones.

The SCM filler was modified using 319 aluminum alloy, a material commonly employed in automobile engines known for its ability to maintain excellent mechanical strength in high-temperature environments. Consequently, tensile tests were conducted under high-temperature conditions of 180 °C and 240 °C in this study, as presented in Table 3. With increasing temperature, the material’s strength decreased, and the total elongation (TE) increased. At 240 °C, the yield strength (YS) of the welded specimen remained over 80% of the YS of the original material, surpassing the weld bead strength of the commercial 5356 aluminum alloy filler after welding. This underscores the ability of the discussed SCM filler to maintain exceptional mechanical properties at high temperatures. Furthermore, the strength of the SCM weld bead exceeded the high-temperature strength of general 6061, regardless of whether 6061 was at 180 °C or 240 °C [40]. This suggests that the SCM filler can fully replace traditional fillers for welding 6061 aluminum alloy in high-temperature conditions.

**Figure 16:**
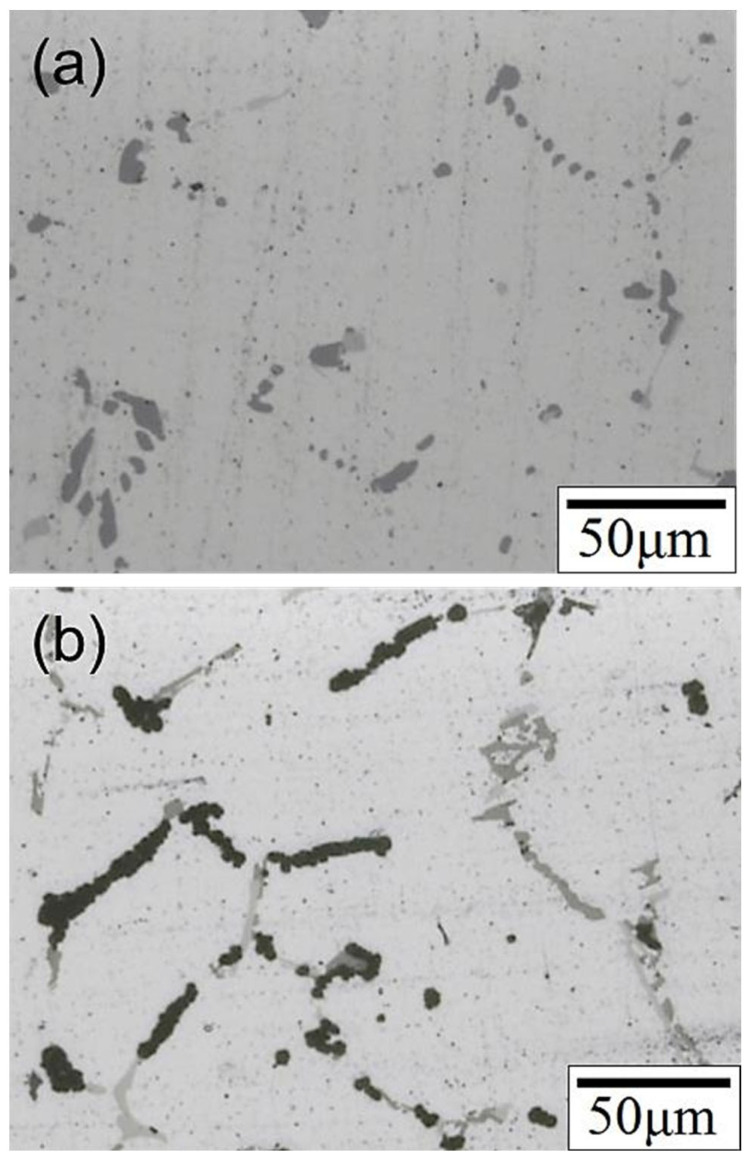
Microstructure of the SCM-T4/T6 specimen: (**a**) before immersion and (**b**) after immersion in 3.5 wt.% NaCl solution.

To investigate the chlorination resistance of the SCM weld bead in this study, the specimen was immersed in a 3.5 wt.% NaCl solution for 1, 3, and 7 days before tensile tests; the relevant results are presented in Table 4. The strength decreased gradually with increasing immersion time. After 7 days of immersion, the strength was still 86% of that of the original specimen, suggesting that the weld bead did not experience a significant deterioration in tensile strength due to chlorination. However, the specimen became brittle after 7 days of immersion. To explore the cause of this phenomenon and understand how saltwater corrodes the SCM weld bead, the SCM-T4/T6 specimen was immersed in a 3.5 wt.% NaCl solution and maintained at 60 °C for 2 days. Figure 16 illustrates that the primary corrosion position is at the secondary phase particles on the grain boundaries. Based on the previously described phase analysis, the grain boundary in the SCM specimen was enriched with Si particles, Al-Fe-Mg-Si, and Al-Cu-Mg-Si phases. These secondary phases may create a potential difference with the Al matrix, facilitating corrosion at the position of the secondary phases [41]. The grain boundary was originally a defect in the material; thus, grain boundary corrosion was the dominant mechanism in this saltwater immersion experiment. It is speculated that after extending the immersion time to 7 days, most of the secondary phases at the grain boundary would be corroded. During stretching, cracks connected in series along the corroded area containing the secondary phase particles, resulting in specimen embrittlement. When 4043 or 5356 fillers are used for 6061 aluminum alloy welding, the extent of corrosion is increased due to the different corrosion potential between the welding fusion zone and the matrix [42], as well as the inability of heat treatment to reduce the percentage of grain boundaries and secondary phase particles [43,44]. The SCM filler developed in this study can alter the specimen’s microstructure and reduce the percentage of grain boundaries and secondary phase particles through applied heat treatment (as shown in Figure 7), thereby decreasing galvanic corrosion and improving corrosion resistance.

Unlike traditional Al-Si fillers, the SCM filler modified in this study contains Cu and Mg, resulting in the precipitation of phases such as Al-Cu-Mg-Si, Al_2_Cu, and Mg_2_Si on the matrix, enhancing the filler’s mechanical strength [31,45]. After heat treatment, Si particles form a eutectic phase with Cu and Mg, further improving the specimen’s hardness and mechanical properties [46,47]. This enables it to maintain favorable high-temperature mechanical properties and corrosion resistance (Table 5). Compared with the traditional ER4043 and ER5356 fillers, the modified SCM filler in this study can undergo heat treatment and has similar mechanical properties to the welded 6XXX aluminum alloy plates after heat treatment; it can maintain the overall strength consistency and address the issue of insufficient welding contact strength. Additionally, the investigation of high-temperature and corrosion properties also verifies that the weld bead exhibits adequate high-temperature strength and corrosion resistance, making it suitable for use in environments with high temperatures and corrosion, such as ships and boilers.

## 4. Conclusions

(1) The microstructure of the weld bead before heat treatment consisted of α-Al dendrites, similar to that of traditional cast A319. After heat treatment, the microstructure remained approximately the same, but the dendrites became larger. The junction of the weld bead and the substrate exhibited a more uniform arrangement.
(2) Before heat treatment, Si particles and some Mg_2_Si phases segregated at the grain boundary. After heat treatment, Si, along with Mg and Cu, overlapped at the grain boundary, indicating that Si elements combined with other elements to form a phase.
(3) After heat treatment, Si particles form a eutectic phase with Cu and Mg, leading to a substantial improvement in the specimen’s strength (from 200 MPa to 320 MPa). This results in no significant difference in strength between the weld bead and the 6061 substrate plate (310 MPa) after welding.
(4) In terms of high-temperature mechanical properties, the UTS of the SCM-T4/T6 specimen was 250 MPa at 240 °C, surpassing the strength of the 6061 substrate plate after T4/T6 heat treatment at high temperatures (approximately 200 MPa).
(5) Heat treatment enabled the SCM weld bead to reduce the percentage of grain boundaries and secondary phase particles (from 3.14% to 1.82%). This reduction effectively minimized galvanic corrosion, allowing the weld bead to maintain adequate strength after being immersed in a 3.5 wt.% NaCl solution for up to 3 days. However, when the remaining grain boundaries and secondary phase particles were preferentially corroded, embrittlement occurred (immersed for 7 days).

## Figures and Tables

**Table 1:** The comparison of the chemical composition of the SCM filler, A319, and 6061 plate.

Element (wt. %)	Si	Cu	Mg	Mn	Fe	Cr	Zr	Sr	Al
SCM	8.80	3.90	0.35	0.26	0.14	0.13	0.08	0.05	Bal.
A319	6.15	3.55	0.05	0.00	0.11	0.00	0.00	0.00	Bal.
6061	0.65	0.30	1.10	0.10	0.40	0.20	0.00	0.00	Bal.

**Table 2:** Tensile properties of the SCM filler and weld zone before and after heat treatment.

	YS (MPa)	UTS (MPa)	UE (%)	TE (%)
Original filler	210 ± 2.8	230 ± 3.0	4.6 ± 0.8	6.9 ± 0.8
Heat-treated filler	321 ± 1.6	376 ± 1.2	7.5 ± 0.5	8.4 ± 0.3
SCM-AW	165 ± 3.1	197 ± 4.2	4.9 ± 1.3	9.1 ± 0.6
SCM-T4/T6	306 ± 2.4	321 ± 2.2	1.3 ± 0.1	3.6 ± 0.3
6061-T6 Standard [36]	276	310	12.0	17.0

**Table 3:** Tensile properties of the SCM-T4/T6 specimen under various temperature conditions.

	YS (MPa)	UTS (MPa)	UE (%)	TE (%)
RT	306 ± 2.4	321 ± 2.2	1.3 ± 0.1	3.6 ± 0.3
180 °C	286 ± 1.1	289 ± 1.7	0.5 ± 0.3	4.8 ± 0.4
240 °C	249 ± 0.4	251 ± 0.4	0.4 ± 0.2	5.3 ± 0.5

**Table 4:** Tensile properties of the SCM-T4/T6 specimen after immersion in 3.5 wt.% NaCl solution for various treatment times.

	YS (MPa)	UTS (MPa)	UE (%)	TE (%)
0-day	306 ± 2.4	321 ± 2.2	1.3 ± 0.1	3.6 ± 0.3
1-day	301 ± 4.1	311 ± 3.2	0.9 ± 0.3	3.1 ± 0.5
3-day	286 ± 0.5	289 ± 0.7	1.2 ± 0.2	4.1 ± 0.1
7-day	269 ± 6.0	269 ± 6.0	0	0

**Table 5:** Comparison of the material properties of a SCM filler and a traditional filler.

	Strength of Filler	Strength of Welded	High Temperature Strength of Welded	Corrosion Resistance	Can Heat Treatment
SCM-filler	✓	✓	✓	✓	✓
Traditional filler	✓			✓	

## Data Availability

Data are contained within the article.
